# Clinical Decision Support Systems (CDSS) for preventive management of COPD patients

**DOI:** 10.1186/1479-5876-12-S2-S9

**Published:** 2014-11-28

**Authors:** Filip Velickovski, Luigi Ceccaroni, Josep Roca, Felip Burgos, Juan B Galdiz, Nuria Marina, Magí Lluch-Ariet

**Affiliations:** 1 Barcelona Digital Technology Centre, 5th floor, 08018 Barcelona, Spain; 2 ViCOROB, Universitat de Girona, Campus Montilivi, 17071 Girona, Spain; 3 1000001 Labs, 08024, Barcelona, Spain; 4 Hospital Clínic, IDIBAPS, Universitat de Barcelona, 08036 Barcelona, Spain; 5 Servicio de Neumología, Hospital Universitario Cruces, 48903 Barakaldo, Bizkaia, Spain; 6 Centro de Investigacíon Biomédica en Red Enfermedades Respiratorias (CIBERES), 07110 Bunyola, Mallorca, Illes Balears, Spain; 7 Departament d'Enginyeria Telemática (ENTEL), Universitat Politécnica de Catalunya (UPC), 08034 Barcelona, Spain

**Keywords:** decision support, COPD, service oriented architecture, integrated care, rule-based systems

## Abstract

**Background:**

The use of information and communication technologies to manage chronic diseases allows the application of integrated care pathways, and the optimization and standardization of care processes. Decision support tools can assist in the adherence to best-practice medicine in critical decision points during the execution of a care pathway.

**Objectives:**

The objectives are to design, develop, and assess a clinical decision support system (CDSS) offering a suite of services for the early detection and assessment of chronic obstructive pulmonary disease (COPD), which can be easily integrated into a healthcare providers' work-flow.

**Methods:**

The software architecture model for the CDSS, interoperable clinical-knowledge representation, and inference engine were designed and implemented to form a base CDSS framework. The CDSS functionalities were iteratively developed through requirement-adjustment/development/validation cycles using enterprise-grade software-engineering methodologies and technologies. Within each cycle, clinical-knowledge acquisition was performed by a health-informatics engineer and a clinical-expert team.

**Results:**

A suite of decision-support web services for (i) COPD early detection and diagnosis, (ii) spirometry quality-control support, (iii) patient stratification, was deployed in a secured environment on-line. The CDSS diagnostic performance was assessed using a validation set of 323 cases with 90% specificity, and 96% sensitivity. Web services were integrated in existing health information system platforms.

**Conclusions:**

Specialized decision support can be offered as a complementary service to existing policies of integrated care for chronic-disease management. The CDSS was able to issue recommendations that have a high degree of accuracy to support COPD case-finding. Integration into healthcare providers' work-flow can be achieved seamlessly through the use of a modular design and service-oriented architecture that connect to existing health information systems.

## Introduction and background

An important problem in healthcare is the significant gap between optimal evidence-based medical practice and the care actually applied. A systematic review [1] of adherence to *chronic obstructive pulmonary disease *(COPD) guidelines by clinicians found that the assessment of the disease and the therapy applied to patients were suboptimal. This situation exists across all chronic-disease care in general: in a multinational survey [2] of chronically ill adults, 14-23% of cases reported at least one medical error in the previous two years.

*Clinical decision support systems *(CDSSs) can be defined as "software that is designed to be a direct aid to clinical decision-making in which the characteristics of an individual patient are matched to a computerized clinical knowledge base (KB), and patient-specific assessments or recommendations are then presented to the clinician and/or the patient for a decision" [3]. CDSSs have the potential to enhance healthcare and health, and to help close the gap between optimal practice and actual clinical care.

The primary objective of the work reviewed in this manuscript is to develop a set of decision-support services so that health professional staff (primary care clinicians and allied health professionals) can obtain fast, reliable and directly applicable advice when dealing with citizens at risk and early-stage patients with COPD, while minimising the impact in work-flows. Specifically, to tackle under-diagnoses, a suite of case-finding services has been developed in order to provide recommendations for both informal (e.g. pharmacy) and formal (e.g. primary care) clinical contexts at early stages of disease development. The case-finding services include a quality-control module to provide recommendations, and expert-quality classifications for forced spirometry tests performed by non-expert clinical providers (primary-care clinicians or allied healthcare providers, such as in a pharmacy) [4,5].

To support the management of disease heterogeneity, decision support services for patient stratification into treatment groups have been designed, relying on three main aspects: firstly, enhancing applicability of well-established rules recommended by the consensus report for the diagnosis, management, and prevention of COPD released by the Global Initiative for COPD (GOLD guidelines) [6]; secondly, using the latest consolidated knowledge on COPD management; and, thirdly, incorporating the knowledge generated by the Synergy-COPD European project, within which the research described in this paper is framed.

## Related work

Ten of the most critical challenges facing the design, development, implementation and deployment of CDSS technology in healthcare were highlighted by a study Sittig et al., 2008 [7]. From these ten "grand challenges", reinforced subsequently by Fox et al., 2010 [8], and relevant to the context of this manuscript are (i) *disseminate best practices in CDS design, development, and implementation*; (ii) *create an architecture for sharing executable CDS modules and services*; (iii) *create internet-accessible clinical decision support repositories*. Furthermore, Kawamoto et al., 2005 [9], performed a systemic review of publications which reported performance of CDSS systems that included description of features. The objective was to determine a correlation between successful CDSS and specific features. They found successful CDSSs had the following three characteristics: (i) *Decision support integrated into the work-flow *; (ii) *decision support delivered at the time and place of decision making *; (iii) *actionable recommendations*.

Another systematic review of CDSSs was performed by Roshanov et al., 2011 [10], with the objective to determine if CDSSs improve the process of chronic care (in diagnosis, treatment, and monitoring) and associated patient outcomes. The authors identified 55 trials that measured and reported the impact of the CDSS on the process of care, and/or patient outcome. Out of the CDSSs that measured the impact on the process of care, 52% demonstrated a statistically significant improvement, and out of the trials that measured patient outcome 31% demonstrated benefits. Specifically, for chronic respiratory diseases (asthma and COPD), only one [11] of the nine reported a positive impact in the process of care: a CDSS for the management of drug therapy in severe asthma. From the five that measured impact on patient outcome, only two [12,13] reported a benefit.

Closely related to our work, Hoeksema et al., 2011 [14] performed a study to report the validity and accuracy of a CDSS designed for the assessment and management of asthma by leading medical institutions in the USA. The system used a similar approach to the Synergy-COPD CDSS by using rules extracted from the guidelines for the diagnosis and management of asthma (EPR-3) [15]. The system assesses the severity of asthma, by applying rules based on a set of inputs, from the patients symptoms, exacerbations, and spirometry (lung function) parameters. Furthermore it recommends the line of treatment, based on the severity level and other factors. The CDSS performed relatively accurately compared to clinicians for the asthma assessment task (pulmonologists agreed with the CDSS 67% of the time, and from the disagreements an expert panel determined that the CDSS was at error 68% of the time, making an overall accuracy level of 78% for the CDSS). The result for the CDSS was poor for the treatment recommendations (pulmonologists agreed with the CDSS 29% of the time, and from the disagreements an expert panel determined that the CDSS was at error 54% of the time, making an overall accuracy level of 62% for the CDSS).

## Methods

### Architecture of the CDSS

The design of the architecture of a CDSS has an important influence on its successful adoption [16]. Four principle architectural models were considered (see also Table 1):

(i) Standalone models: this architecture was used by early CDSSs. Since it has no integration to an external *health information system *(HIS) or *electronic health record *(EHR), it requires the user to enter all findings and clinical information, thus being time consuming. The advantage of such systems is that they are easily sharable and transferable to different centres (i.e. just by copying the software across).

(ii) Integrated models: this architecture is tightly coupled to the HIS or EHR. Such CDSSs may be proactive in issuing alerts, and make less input demands on users as the data are already available. The disadvantage of such a model is the difficulty to be shared, as it is dependent on vendor specific HIS or EHR;

(iii) Standard-based models: this architecture separates the CDSS from the HIS and the EHR. Interoperability is achieved through a standardization of the computerized representation of clinical knowledge through the use of *computer interpretable guidelines *(CIGs) [17-20].

(iv) Service-oriented models: this architecture (e.g., [21,22]) separates the CDSS from the HIS, but integrates them using standardized, service based interfaces. The interface encodes the clinical data and recommendations in a formal representation using ontologies and vocabularies. Thus, standardization is based on the data transferred between the HIS and CDSS instead of the the guidelines and clinical rules executed by the CDSS as in standard-based systems.

**Table 1:** Comparison of features in CDSS architectures.

Architectural model's feature	Stand-alone	Integrated	Standard-based	Service-oriented
Service transferable across clinical centres	Yes	No	Yes	Yes
Manual data-entry to CDSS minimized	No	No	Yes	Yes
Connected to EHR or HIS	No	Yes	Yes	Yes
Vendor independent EHR or HIS	N/A	No	Yes	No
Standardized clinical knowledge representation	No	No	Yes^†^	Sometimes
Standardized clinical data representation	No	No	Sometimes	Yes

^† ^despite an on-going effort for the last two decades, there is still not a widespread adoption of standard-based systems, nor a widespread CIG format

See [16] for an extensive review.

**Figure 1:**
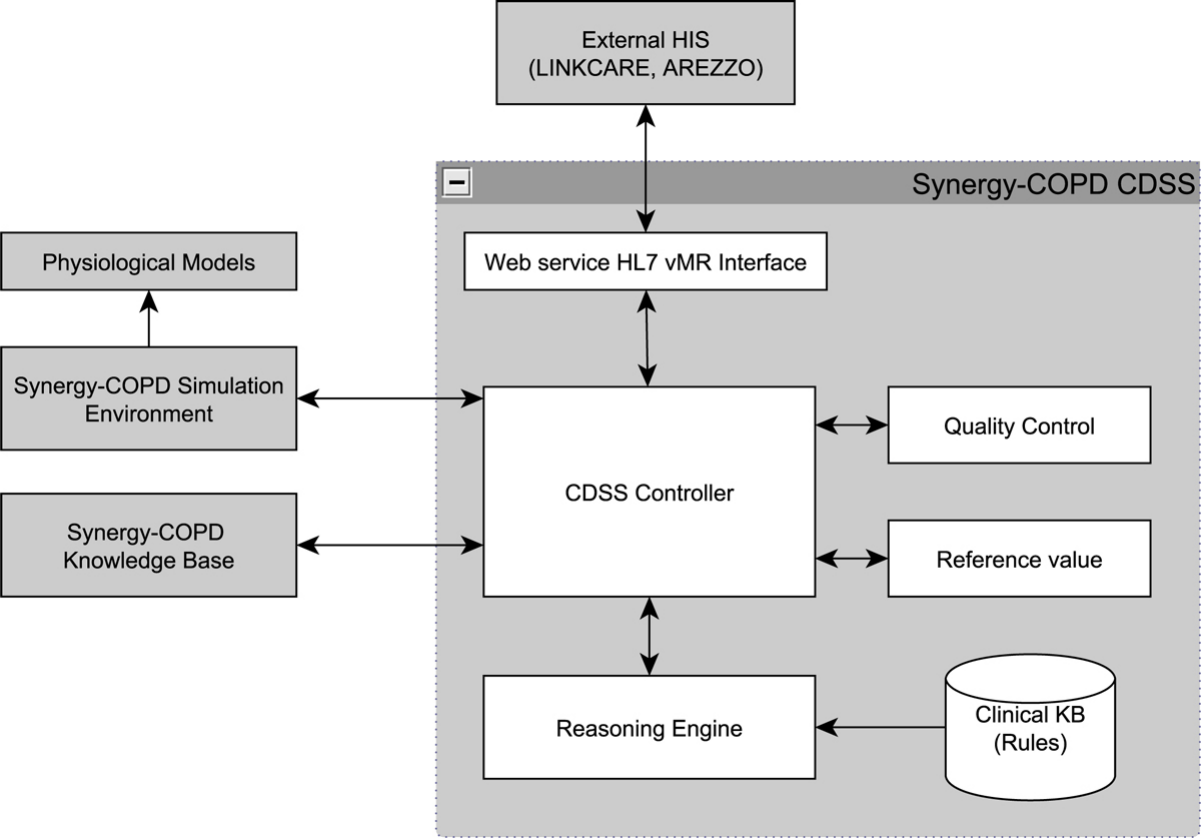
**CDSS architecture depicting internal modules, external user HIS, and external supporting Synergy-COPD systems**.

A service-oriented approach was selected for the CDSS as it covered the most critical features as summarized in Table 1. In this model the CDSS is interfaced through a web service protocol, with clinical data being exchanged through an interoperable format described later in the section on clinical data representation. The diagram in Figure 1 illustrates the architecture, showing the main interfaces between the external user systems and its internal components, which are described as follows.

#### Controller

The CDSS Controller is responsible for coordinating all communication between internal components and external systems during the execution of a decision support task. It manages user requests/responses that contain clinical data from the patient communicated in the HL7 *virtual medical record *(vMR) format, the running of the reasoning engine, the quality-control module, and the reference-value module.

#### Reasoning engine and clinical knowledge base

CDSSs may be classified by the reasoning or inference methods they use. These methods, along with demonstrated implementations for COPD management, are listed in Table 2. Approaches that explicitly model knowledge are preferred in the clinical domain because they facilitate the often-needed justification of the recommendation. The work-flow driven approach, by way of encapsulating clinical care protocols into computer interpretable guidelines, has been demonstrated by *J. Fox *and his team successfully through the PROforma language [23,24]. In Synergy-COPD, a rules-based reasoning paradigm was adopted for the CDSS. This approach was to complement existing HISs (Linkcare and Arezzo Pathways) that already implemented clinical work-flows, thus focusing on the critical clinical decision tasks in COPD management, modelled as production rules.

**Table 2:** Inference methods used in CDSS

Method	Description	Implementations
Work-flow driven ^1^	Logical flows contain statements that reference and manipulate clinical data, usually executed in a serial manner, with control structures that direct the flow of decision making through the procedure.	[23,24]
Rules-based reasoning ^1^	Medical knowledge is captured through a collection of IF-THEN expressions. Reasoning by forward chaining (the most common one) links rules together until a conclusion is reached.	[50-52]
Probabilistic reasoning ^1,2^	Bayesian networks and graphical representation that describes the causal relationships between diseases and symptoms with conditional probabilities.	[53]
Machine learning (ML) ^2^	Machine learning and statistical techniques, by learning or training, are used on existing, large datasets of clinical data.	[54,55]

^1^Clinical knowledge explicitly modelled

^2^Clinical knowledge derived or learnt from data of past cases

Rule-based programming has its foundations in symbolic production systems, and its basic approach is to decompose a computation into a set of elementary transformations, embodying, in the case of this research, clinical tasks. Each elementary transformation attempts to match its input against a set of templates, and, if some of these match, a rule corresponding to one of the templates is chosen, and the action associated with the rule is executed. Most rule-based inference engines use the Rete algorithm [25]. To represent rules, the CDSS uses the open-source Java-based *JBoss Drools *[26], which has an easier-to-interpret syntax than representations used by competing systems: CLIPS [27] and Jess [28].

**Figure 2:**
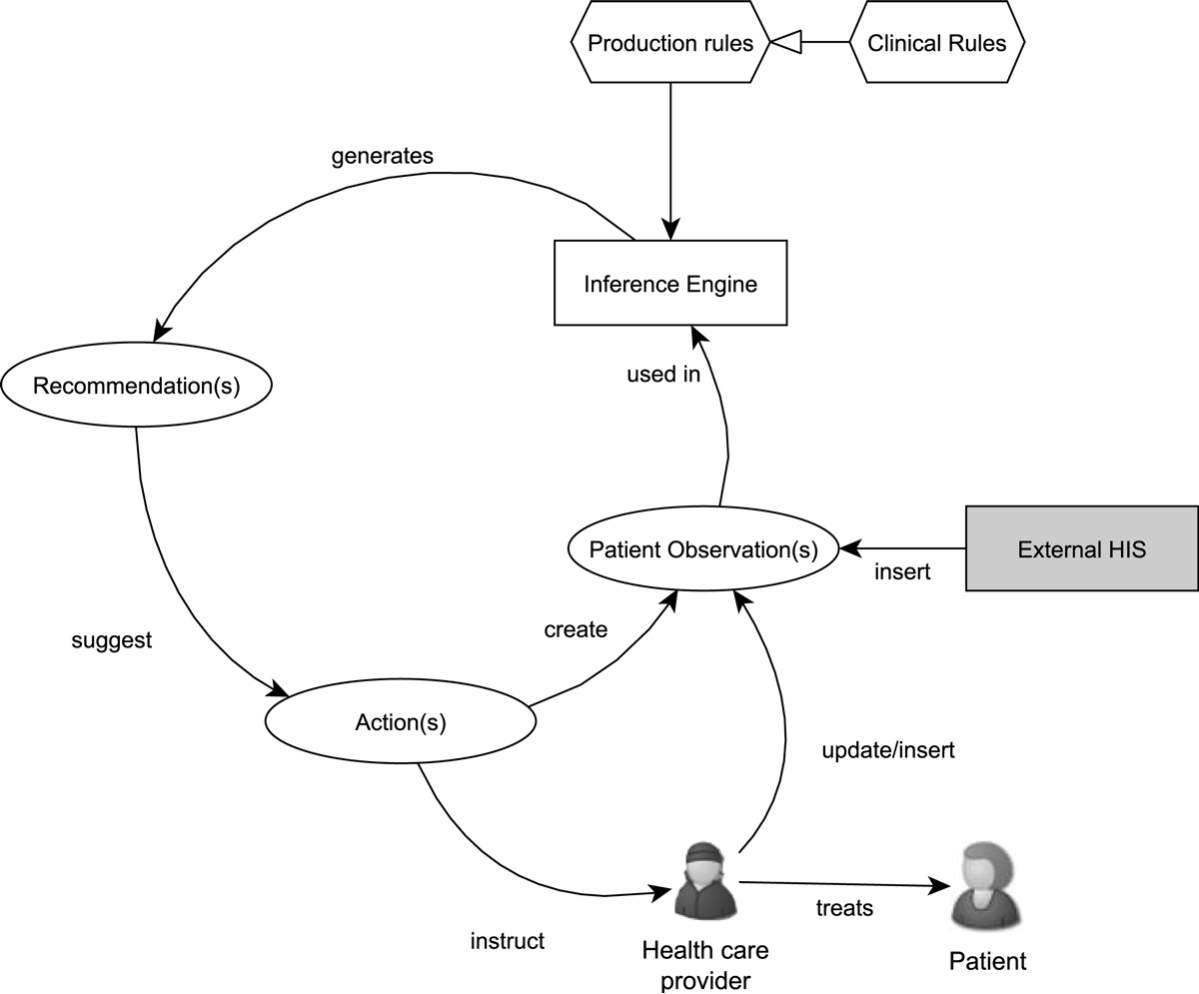
**Reasoning paradigm**.

Figure 2 is an illustration of the reasoning paradigm implemented by the CDSS. The rule-based engine operates on inserted facts about a patient that are transmitted to the CDSS by the external HIS requiring decision-support services. Facts may be particular clinical findings or measurements or demographic information about the patient (e.g. "forced vital capacity = 3.7 L"; "dyspnea's MRC severity grade = 4"; "gender = male"). Rules represent mathematical or logical knowledge that infers (produces) new facts from currently available facts. Clinical rules are a subclass of rules that represent clinical and medical knowledge that infers new facts or medical recommendations from currently available medical facts. Clinical rules operate within a modular context that allows, at any particular moment, firing only the specific set of rules associated with the specific clinical task at hand (e.g. case-finding, diagnosis, assessment).

In this paradigm, the clinician has to ultimately take the final decision. The CDSS generates recommendations based on the patient's personal profile; each recommendation specifying a recommended course of action for the clinician (e.g. "Diagnose patient with COPD.") and the reason why this is the case (e.g. "Symptoms consistent with COPD according to GOLD guidelines' criterion: FEV1/FVC *<*0.7"). If a recommendation is accepted, it may either automatically create new facts into the system augmenting the patient's medical profile (e.g. COPD added to the list of diseases), or instruct the clinician to perform further actions (e.g. "Take a spirometry measurement after applying a bronchodilator and report back the results.").

#### Quality control module

The quality-control module implements an algorithm for assessing the acceptability of an individual forced spirometry manoeuvre. The automatic validation of the spirometry measurements consists of identifying wrong/flawed tests, or acceptable/valid tests. Hence an indication is provided regarding the quality of the measurements performed, and feedback or indication is provided regarding the reliability, or confidence level, of the manoeuvre or set of manoeuvres. This is used by an evaluator to assess the quality of a full spirometry test comprising more than one manoeuvre. No expert intervention is necessary and support can be provided in a clinical setting where the clinician or healthcare provider is not an expert in spirometry tests.

#### Reference value module

The reference-value module invokes continuous prediction equations and their *lower limit of normal *(LLN) for clinical parameters - specifically, it uses spirometric reference values specified by Hankinson et al., 1999 [29] and Quanjer et al., 2012 [30] for case-finding and diagnosis.

#### External supporting Synergy-COPD systems

The interfaces to Simulation Environment [31,32] and Synergy-COPD Knowledge Base [33,34] (that host the predictive models [35-38] developed within the Synergy-COPD project) have been developed for prognostic extensions to the CDSS. Furthermore the Synergy-COPD Knowledge Base is accessed for drug-drug interaction data.

### Clinical data representation

The service-oriented architecture allows the CDSS to deliver decision support capabilities to any external HIS that is able to provide the input clinical data of the patient and receive as output clinical recommendation through a well specified Simple Object Access Protocol (SOAP) interface defined in the web services description language (WSDL). The underlying format that was selected to contain the input clinical data was the HL7 virtual medical record (vMR) [39,40]. The vMR is a data model for representing clinical data specifically optimised for decision support tasks; it captures data about the patient's demographics, clinical history, and is also designed to capture CDSS-generated recommended actions such as suggested clinical interventions, therapies, procedures, and assessments. Data in the vMR are represented using user-defined vocabularies; to enhance interoperability, standardised vocabularies with clear semantics were used within the vMR messages to encode clinical concepts. The vocabularies are shown in Table 3, which includes an example concept and the associated code.

**Table 3:** Standardised vocabulary used in clinical data exchange.

HL7 vMR item	Vocabulary	Example (Code)
Observation	SNOMED-CT [56]	forced vital capacity (50834005)
Procedure	SNOMED-CT	spirometry test (127783003)
Problem (Disease)	ICD-10 [57]	COPD (J44)
Ethnicity	Ethnicity - CDC [58]	white (2106-3)
Language	ISO 639 language code [59]	English (en)

### Development

**Figure 3:**
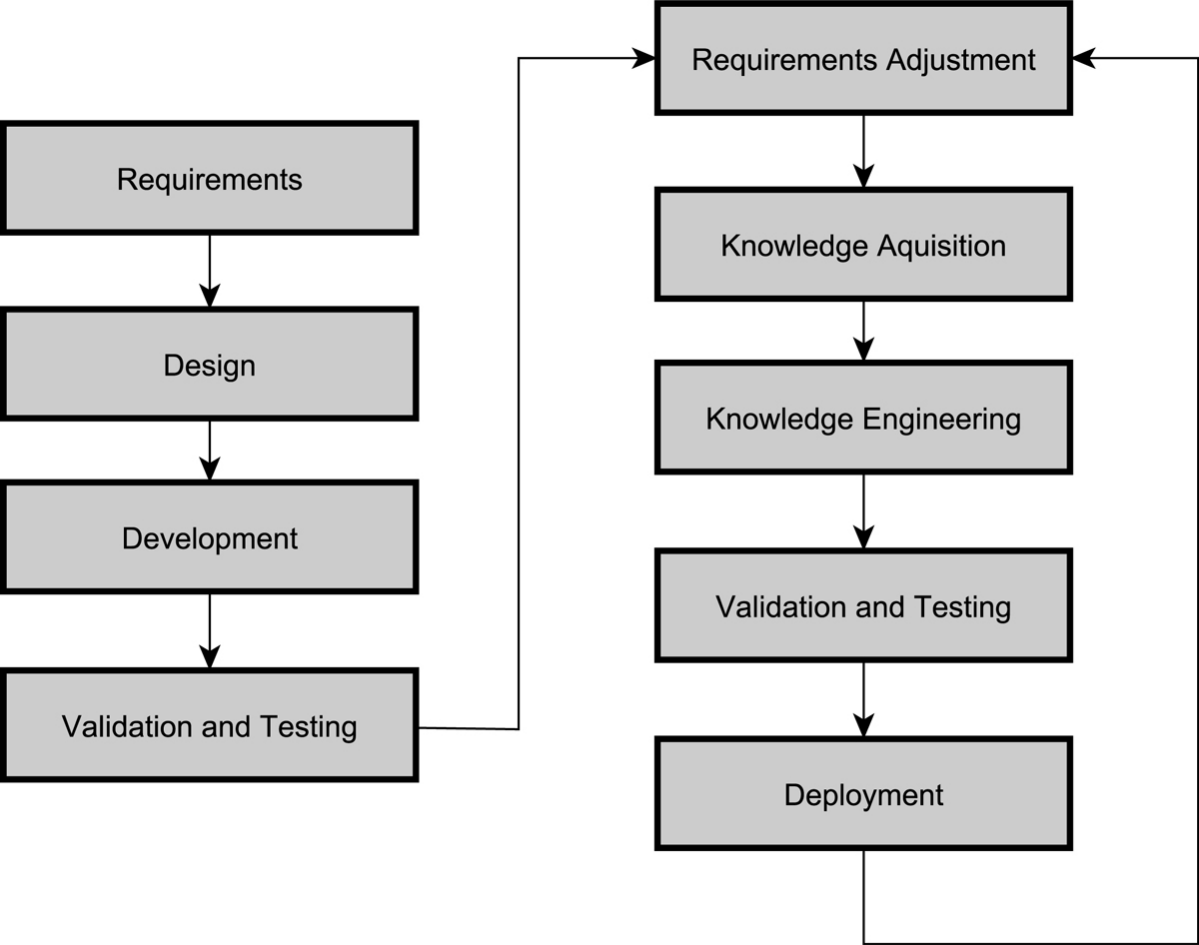
**Adapted incremental software development model for the CDSS**.

The CDSS was constructed using an iterative and incremental development model adapted from [41]. Figure 3 shows the main development phases. After the initial requirements specification, and design phase, the framework containing the main CDSS components was developed. Three incremental cycles were completed to develop the CDSS web services, and within each cycle the following phases were executed:

(i) *requirements adjustment *- functionalities for subsequent clinical task refined;

(ii) *knowledge acquisition *- clinical guidelines interpreted by respiratory specialists and defined as rules or as algorithm;

(iii) *knowledge engineering *- translation of clinical rules into Drools rules representation and classification into specialized CDSS modules (quality control, reference value);

(iv) *validation and testing *- input test cases and expected output defined and tested against CDSS web services;

(v) *deployment *- secure web service interface exposed and integrated into an existing HIS platform.

## Results

Several decision-support web-services were deployed in a secured environment online for preventive management of COPD patients with performance benchmarked. The web services were incorporated into two existing HISs: all web services into the Linkcare platform [42] and the spirometry quality control web service into Arezzo Pathways [43].

### Decision support web services

#### Spirometry quality control

This service, through spirometry-test results consisting of a set of raw signals from spirometry manoeuvres, determines: quality grade (A, B, C, D or F) of the spirometry test; best lung function parameters for the volume that has been exhaled at the end of the first second of forced expiration (FEV1), the vital capacity from a maximally forced expiratory effort (FVC), the highest forced expiratory flow (PEF), back extrapolated volume (BEV), and their associated manoeuvres; acceptability of each manoeuvre; ranking of each manoeuvre; for manoeuvres deemed to unacceptable, the reasons for their rejection.

#### case-finding: Eligibility for spirometry test

Through an inclusion/exclusion criteria, represented as Drools rules in the clinical knowledge-base, this service generates advice on subjects at risk of COPD. Subjects are selected for further investigation based on demographics, risk factors, and symptoms. The system produces patient-specific advice for: eligibility of the subject for a further spirometry test; recommendations for smokers based on their dependency.

#### Case finding: Preliminary evaluation

From the results of a pre-bronchodilation spirometry of an eligible subject, this service determines: requirement to refer the subject to primary care for further tests; preliminary evaluation of lung function; cessation advice for smokers based on their dependency.

#### Diagnosis: Primary care evaluation

From a patient's full exam consisting of pre-bronchodilation and post-bronchodilation spirometry, this service determines probable COPD cases, evaluates lung function, and issues cessation advice for smokers based on their dependency.

#### Assessment: Patient stratification

From a patient's post-bronchodilation spirometry result and index scores from standard questionnaires (COPD assessment test [44], modified Medical Research Council dyspnea scale [45]) the patient is stratified into the GOLD 2011 [6] categories: group A - low exacerbation risk, less symptoms; group B - low exacerbation risk, more symptoms; group C - high exacerbation risk, less symptoms; group D - high exacerbation risk, more symptoms. Each stratification group has an associated recommended set of pharmacological and non-pharmacological therapies.

### Evaluation of the CDSS as a diagnosis service

#### Validation dataset

The performance of the CDSS diagnosis service was compared with an anonymised database of patients from Primary Care centres participating in forced-spirometry training in a web-based remote support program to enhance quality of forced spirometry done by non-expert professional in the Basque Country region of Spain. Forced-spirometry testing was done using a *Sibel 120 SIBELMED *spirometer. The spirometry quality and diagnosis evaluation was done by one respiratory specialist. Inclusion criteria to form the validation data set were:

(i) age of the patient greater than or equal to 40;

(ii) forced spirometry taken and recorded as an electronic record before and after the application of bronchodilators;

(iii) respiratory specialist used option menu to select the appropriate diagnosis (rather than entered through the free text field).

After applying the inclusion criteria, the validation set was formed containing 323 cases. The use of the dataset for validation purposes was approved by the Ethical Committee of the Hospital Clinic í Provincial de Barcelona.

#### Benchmarking the diagonisis service

The clinical data for each case in the validation set was fed into the CDSS diagnosis service, the result was compared against the respiratory specialist classification of the case. The mapping in Table 4 was used to compare the specialist classification to the CDSS for the purposes of validation.

**Table 4:** Mapping from respiratory specialist classification to CDSS diagnosis classification.

Specialist class	CDSS class
Normal, no obstruction pattern	Unlikely COPD
Mild, obstruction pattern	Likely COPD
Moderate obstruction pattern	Likely COPD
Severe obstruction pattern	Likely COPD

Sensitivity and specificity of the CDSS were calculated for cases in the validation set classified as Likely COPD or Unlikely COPD.

(1) $$sensitivity=\frac{TP}{TP+FN}$$

(2) $$specificity=\frac{TN}{TP+FP}$$

wherein TP (true positive) corresponds to cases classified as *Likely COPD *by both CDSS and the specialist; TN (true negative) corresponds to cases classified as *Unlikely COPD by the CDSS *and the by the specialist; FP (false positive) indicates cases classified as *Likely COPD *by the CDSS, but classified as class *Unlikely COPD *by the specialist; and, FN (false negative) corresponds to cases classified as *Unlikely COPD *by the CDSS, but as *Likely COPD *by the specialist. The CDSS produced 101 diagnosis recommendations as likely COPD, and 222 recommendations as unlikely COPD. 297 cases correctly matched the assessment of the specialists (92%). Sensitivity and specificity calculations were calculated to be 90% and 96%, respectively. Table 5 shows the details of these results as a confusion matrix.

**Table 5:** Confusion matrix of diagnosis

		Specialist diagnosis	
		**Likely COPD**	**Unlikely COPD**
	
CDSS Diagnosis	Likely COPD	78	23

	Unlikely COPD	3	219

## Integration

The CDSS operates by receiving and sending standardized messages, and relies on an existing HIS to present its recommendations to the healthcare professional on screen or via the issuance of a report. Two such HISs have successfully implemented the CDSS web services. The CDSS response time for all decision support services was acceptable (within seconds) to the clinical task at hand, and thus allowed a seamless integration into the existing HIS.

### Linkcare

Linkcare is an integrated-care open platform allowing healthcare professionals (specialists, general practitioners, case managers, nurses, etc.) to share clinical knowledge around a patient centric model. A Linkcare mobility module allows posting activities to be performed by patients, using their smart-phone, tablet or a web portal. Such activities include follow-up questionnaires and medical measurements, such as measurements by pulse-oximeters, glucometers and spirometers, and measurements of blood pressure. Healthcare professionals can exchange care protocols and clinical data around Integrated Practice Units or specific Clinical Research teams. Integrating the CDSS web services with the Linkcare platform allows healthcare professionals to be assisted in making clinical decision relating to case-finding, diagnosis, and stratification of COPD patient.

### Arezzo Pathways

Arezzo Pathways combines best practice clinical guidelines with individual patient data to dynamically generate care pathways and provide decision recommendations specific to each patient at the point of care. This assists clinicians in managing patients with long-term conditions and in making timely and appropriate referrals. The CDSS web service offering spirometry quality-control and quality-assurance has been integrated into Arezzo Pathways.

### Communication protocol

**Figure 4:**
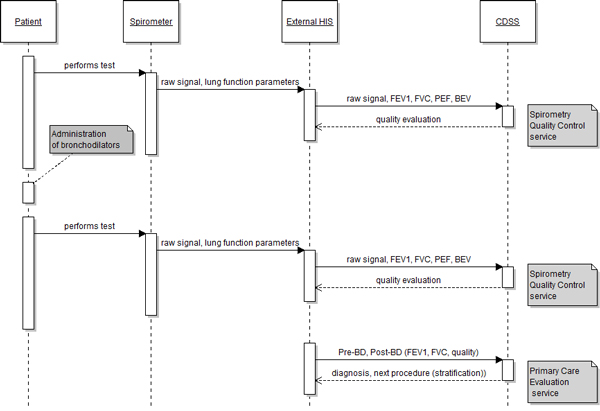
**System interaction during confirmation of a COPD diagnosis in a primary care setting**.

Figure 4 shows the use of the CDSS through a primary care scenario and the exchange of messages between Linkcare and the CDSS web services, with the objective for a clinician to confirm COPD in a patient. The clinician uses the Linkcare platform to enter the details of the patient in the system, or retrieve them from the EHR. The patient has already been assessed as being at risk of COPD, and the primary care clinician needs to confirm the COPD. For this, the primary care clinician needs to perform a full spirometry exam, i.e. two tests: one before the application of bronchodilators, and one after. To ensure the measurement taken with the spirometer satisfies criteria for an acceptable and reliable test, the full sampled signal, along with the lung function parameters are sent as two request messages, one for each test (pre and post-bronchodilation), to the Spirometry Quality Control web service. Linkcare uses the Diagnosis - Primary Care Evaluation web service to support the clinician in the decision of the diagnosis of the patient. The Linkcare platform sends the CDSS a request message with details of the lung function parameters obtained during the spirometry measurement. The CDSS replies with a response containing the evaluation to confirm the diagnosis and a recommendation to schedule an appointment for further evaluation and stratification.

## Discussion and conclusion

A large epidemiological report on the prevalence and burden of respiratory disorders carried out in the general population of Catalonia [46] stresses two important facts in relation to COPD: (i) There is high prevalence in the population greater than 65 years of age (36% in men and 22% in women); (ii) there is a significant level of under-diagnoses (76%). Moreover, in the UK, over 25% of people with a diagnostic label of COPD have been wrongly diagnosed, usually because of poorly-performed spirometry [47]. This research addresses the above issues by targeting the identification of occult COPD cases aiming at a better delineation of the natural history of the disease. The CDSS services for detection and diagnosis provide this capability, and an initial validation of the diagnostic potential of the CDSS shows promising results (overall accuracy of 92%) in the ability to provide high quality recommendation service for the diagnosis of COPD.

The *Global Initiative for Chronic Obstructive Lung Disease *(GOLD) consensus report released initially in 2011 *Global Strategy for the Diagnosis, Management, and Prevention of COPD *[6] recommended a major revision in the management strategy for COPD. An updated report released in January 2014 maintains the same treatment paradigm. Assessment of COPD is based on the patient's level of symptoms, future risk of exacerbations, the severity of the spirometric abnormality, and the identification of co-morbidities. This assessment has a limited practical applicability because of its complexity. To facilitate the adoption of the new GOLD classification, it has been incorporated as clinical rules into the clinical knowledge-base, and deployed as a CDSS service. And because an increasing number of reports indicate that the new GOLD classification is not providing added value in terms of clinical impact [48], future activities will be devoted to the development of richer stratification schemes that enrich the assessment capabilities of the CDSS using existing knowledge that is not incorporated in current schemes (i.e. information about general health status, disease severity, activity level, co-morbidities and use of healthcare resources), and by including new knowledge acquired in the Synergy-COPD European research project.

Another revision in the GOLD report was spirometry changed from being a supportive diagnostic tool, to be a requirement for the diagnosis of COPD. This has produced a strong need to support spirometry testing carried out by non-specialized professionals in primary care and allied health providers. This need is addressed through the spirometry quality control CDSS service capable of near expert level feedback on forced-spirometry manoeuvres. An article focusing on the module and performance of the quality control service is to appear in the Journal of Medical Internet Research [49].

Finally the research we present confronts the challenges and applies the characteristics that were originally highlighted in the related work. Firstly, it demonstrates through the modular design and service-oriented architecture of the CDSS framework, the capability of making available internet accessible decision-support modules and services shareable by multiple external HIS platforms. Furthermore the CDSS is able to be directly embedded into the user's work-flow by integration into existing HIS platforms with recommendations *generated at the time and place of decision making*.

### Limitations

We acknowledge three principle limitations of the study. Firstly, only data from one respiratory expert was used as ground truth for comparison to the CDSS recommendation in the evaluation. Ideally further independent validation, involving a panel of experts would be more robust in evaluating CDSS performance. Secondly, although our design allows for multiple HIS distributed across the world to use the single CDSS specialised in COPD, we acknowledge guidelines in diagnosis, assessment, and treatment will differ across national borders to suit specific population. The CDSS's modular design allows for instances of the CDSS to be deployed that cater for the specific medical policy or protocol, only by modification of the rules. Thirdly, although a CDSS may achieve a high degree of accuracy and performance, the impact of when it is deployed in an actual healthcare setting needs to be assessed separately before plans for large scale deployment are developed. As part of this deployment process, the current version of the CDSS is going through a qualitative evaluation using a focus group approach that includes: primary care physicians, nurses, pharmacists and respiratory specialists. A protocol to assess the clinical impact of the use of the CDSS is to be initiated.

## Conclusion

Specialized decision support can be offered as a complementary service to existing policies of integrated care for chronic-disease management. The current research has generated a CDSS capable of addressing important issues facing COPD management in case-finding, diagnosis and stratification. The CDSS is able to issue recommendations that have a high degree of accuracy to support COPD case-finding. Moreover, integration into healthcare providers' work-flow has been demonstrated through the use of a modular design and service-oriented architecture that connect to existing health information systems already in use.

## Competing interests

The authors declare that they have no competing interests.

## Authors' contributions

FV and LC conceived and designed the initial CDSS. FV was the lead developer and research engineer of the CDSS. JR and FB provided clinical expertise for the design of the COPD rules in the case-finding, assessment, and diagnosis services. FV and JR supported the deployment of the CDSS into existing health information systems. MLA contributed to design and requirement refinements in the final stages and iterations of the CDSS development. FV, JBG and NM were involved in the evaluation of the diagnostic performance of the CDSS. FV and LC wrote the first draft of the manuscript. FV, LC, JR, FB, JBG, NM, and MLA contributed to the writing of the manuscript. FV, LC, JR, FB, JBG, NM, and MLA agree with manuscript results and conclusions. LC: part of this work was completed while at Barcelona Digital Technology Centre

## Declaration

Publication of this article has been funded by the Synergy-COPD European project (FP7-ICT-270086). The opinions expressed in this paper are those of the authors and are not necessarily those of Synergy-COPD project's partners or the European Commission.

This article has been published as part of *Journal of Translational Medicine *Volume 12 Supplement 2, 2014: Systems medicine in chronic diseases: COPD as a use case. The full contents of the supplement are available online at http://www.translational-medicine.com/supplements/12/S2.
